# Cerebral infarction associated with benign mucin-producing adenomyosis: report of two cases

**DOI:** 10.1186/s12883-018-1169-2

**Published:** 2018-10-04

**Authors:** Koki Okazaki, Fumiaki Oka, Hideyuki Ishihara, Michiyasu Suzuki

**Affiliations:** Department of Neurosurgery, Yamaguchi University School of Medicine, 1-1-1 Minami-Kogushi, Ube, Yamaguchi, 755-8505 Japan

**Keywords:** Cerebral infarction, Adenomyosis, Trousseau’s syndrome, Benign tumor, Case report

## Abstract

**Background:**

Cerebral infarction associated with a malignant tumor is widely recognized as Trousseau syndrome. In contrast, few cases of cerebral infarction associated with benign tumors have been reported. We present two cases of embolic stroke that seemed to be caused by mucin-producing adenomyosis.

**Case presentation:**

The patients were women aged 42 and 50 years old. Both patients developed right hemiparesis and aphasia, and cerebral infarctions were detected in the left cerebral hemisphere. There were no other abnormal findings, except for elevation of CA125 and D-dimer. Trousseau syndrome was suspected in both cases, but whole body examinations did not reveal any malignant tumors. However, uterine adenomyosis was detected in both patients.

**Conclusions:**

From our findings and a review of the literature, both mucin-producing malignant tumors and mucin-producing benign tumors such as adenomyosis may cause hypercoagulability and cerebral infarction. This mechanism should be considered in a case of a young to middle-aged woman with embolic stroke of an undetermined origin.

## Background

Mucin-producing malignant tumors may cause hypercoagulability and associated cerebral infarction that is widely referred to as Trousseau syndrome. Adenomyosis is also reported to produce mucin and to cause hypercoagulability [1, 2]. Here, we present two cases of embolic stroke that developed in middle-aged women and seemed to be caused by benign mucin-producing adenomyosis.

## Case presentation

**Fig. 1:**
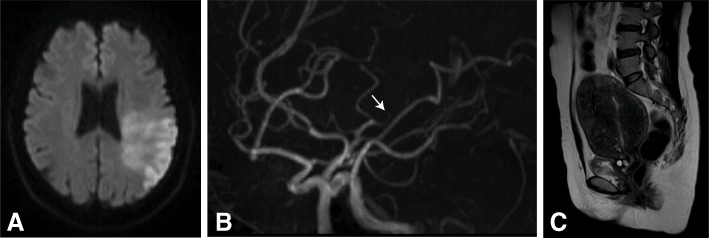
**a** Diffusion-weighted magnetic resonance imaging (MRI) revealed an infarct in the left middle cerebral artery territory. **b** Magnetic resonance angiography showed occlusion at left M2 (arrow). **c** T2-weighted pelvic MRI revealed enlargement of the uterus and obscure junctional zone, suggesting adenomyosis

Patient 1 (Fig. 1): A 42-year-old woman with no medical history of note presented with right hemiparesis and aphasia, and was admitted to our hospital. The actual onset time was unknown. On arrival, her National Institutes of Health Score Scale (NIHSS) was 20. Diffusion-weighted brain magnetic resonance imaging (MRI) showed a hyperintense signal in the left middle cerebral artery (MCA) territory, and MR angiography (MRA) indicated occlusion of the left superior M2 (Fig. 1a, b). Because the infarct area seemed to match with the occluded artery territory, reperfusion therapy was not performed. After admission, we performed examinations to investigate the cause of cerebral infarction. Transthoracic echocardiography (TTE) and transesophageal echocardiography (TEE) showed no remarkable findings. A 24-h Holter electrocardiogram (ECG) did not show atrial fibrillation or other arrhythmia. Carotid echography and carotid MRA did not show atherosclerotic changes at proximal arteries. Blood tests were conducted to investigate the possibility of coagulation disorders, such as antiphospholipid antibody syndrome, collagen disease, protein S and C deficiency, antithrombin III deficiency, and tumor markers. However, the results were unremarkable, except for elevation of D-dimer (1.4 μg/mL) and CA 125 (395 U/mL; normal, < 35 U/mL). Whole body enhanced computed tomography (CT) revealed no malignancy. Pelvic MRI showed uterine adenomyosis (Fig. 1c).

**Fig. 2:**
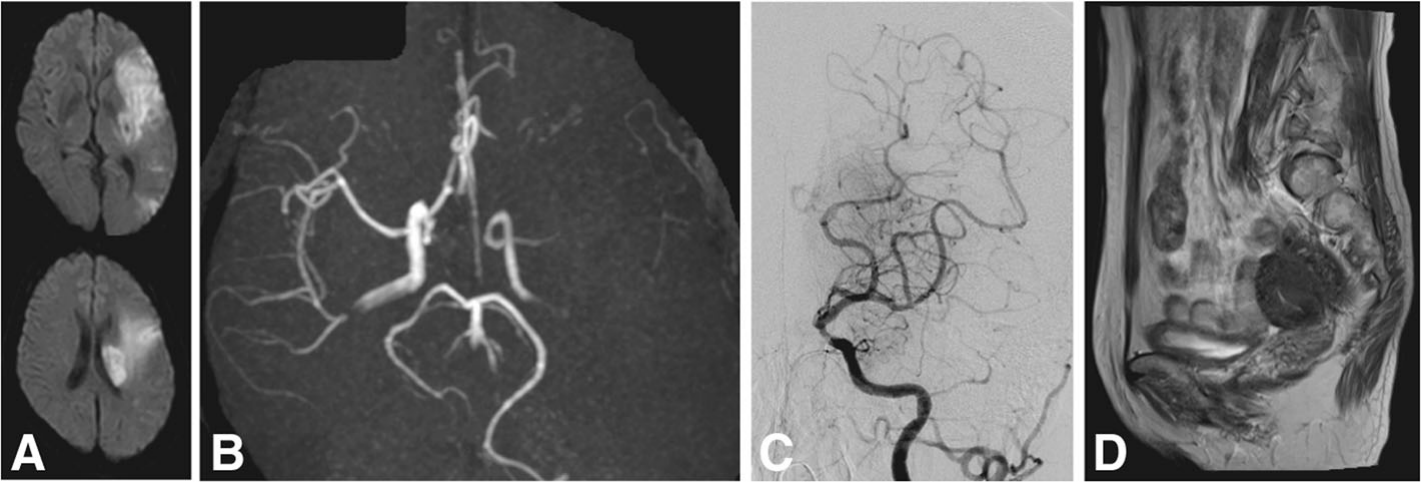
**a** Diffusion-weighted MRI revealed an infarct in the left middle cerebral artery territory. **b** Magnetic resonance angiography at a previous hospital showed left M1 occlusion. **c** Angiography revealed partial recanalization of the left middle cerebral artery. **d** Pelvic MRI revealed adenomyosis

Patient 2 (Fig. 2): A 50-year-old woman with no medical history of note presented with right hemiparesis and mixed aphasia, and was admitted to a local hospital. The onset time was unknown. Diffusion-weighted imaging (DWI) in brain MRI revealed a hyperintense area in the left MCA territory. MRA showed occlusion at M1 (Fig. 2a, b). The patient was referred to our hospital for further examination and treatment. On arrival, her NIHSS was 23. Emergent digital subtraction angiography (DSA) was performed and partial reperfusion of the left MCA was found (Fig. 2c). We hesitated to perform endovascular treatment because of the large infarction. After admission, we performed examinations to investigate the cause of cerebral infarction. TTE and TEE showed no remarkable findings, and a 24-h Holter ECG did not show atrial fibrillation or other arrhythmia. DSA and carotid echography did not show atherosclerotic changes at proximal arteries. Blood tests performed to investigate the presence of coagulation disorders (as listed above for case 1) were unremarkable, except for elevation of D-dimer (3.7 μg/mL) and CA125 (143 U/mL; normal, < 35 U/mL). Whole body enhanced CT revealed no malignancy. Pelvic MRI showed uterine adenomyosis (Fig. 2d). Her aphasia gradually improved, but motor aphasia remained.

Based on the above findings, both cases were finally diagnosed with cerebral infarction due to Trousseau syndrome-like hypercoagulability associated with adenomyosis. For secondary prevention, the first patient was treated with warfarin and the second patient was treated with rivaroxaban, and there has been no recurrence for 68 and 19 months and modified rankin scale is 1 and 4, respectively.

## Discussion

The risk of thrombotic complication is high in patients with malignant tumor, and this condition is referred to as Trousseau syndrome [3]. Varki reported multiple mechanisms of hypercoagulability in patients with malignant tumor, involving tissue factor, mucin, cysteine protease, and various cytokines [4]. Especially, mucin promotes platelet aggregation by interaction with platelet P-selectin and leukocyte L-selectin, with resulting hypercoagulability [5]. CA125 is a repeating peptide epitope of mucin MUC16 and a marker of mucin-producing malignant tumors such as ovarian cancer [6]. Elevation of CA125 in patients with a malignant tumor increases the risk of ischemic stroke [7–9]. Hypercoagulability and elevation of CA125 in patients with adenomyosis has also been reported [1, 2], and as for patients with cancer, hypercoagulability can occur in patients with adenomyosis due to increased expression of tissue factor [2]. Indeed, as shown in Table 1, elevation of D-dimer at onset has been found in all except one of the reported cases of ischemic stroke related to adenomyosis. Elevation of CA125 was also detected in both of our cases. The previous and current cases indicate that adenomyosis itself seems to cause hypercoagulability through a mechanism similar to that of Trousseau syndrome and may cause ischemic stroke. In contrast to previous reports, both of our patients had large vessel occlusion with emboli and large infarction. As for patients with Trousseau syndrome, both multiple infarction and large vessel occlusion can also occur in patients with mucin-producing adenomyosis and could cause severe neurological deficits, as shown in our cases.

**Table 1:** Summary of cases of ischemic stroke related to adenomyosis

Case No. [Ref]	Age (y.o)	CA125 (U/mL)	D-dimer (μg/mL)	Secondary prevention	Recurrence
1 [9]	45	159	1.1	Antiplatelet, GnRH agonist	(−)
2 [9]	44	Not mentioned	FDP 5.9 μg/mL	Warfarin, GnRH agonist	(−)
3 [9]	55	42.6	0.57 (normal)	Aspirin, GnRH agonist	(−)
4 [8]^a^	42	1750	6.0	Antiplatelet (6 m). GnRH agonist (6 m)	(+)
5 [9]^a^	42	907	4.1	Warfarin, GnRH agonist	(−)
6 [11]	59	334.8	7.0	Discontinuation of hormone replacement therapy	(−)
7^b^	42	395	1.4	Warfarin	(−)
8^b^	50	143	3.7	Rivaroxaban	(−)

^a^Case Nos. 4 and 5 are the same patient

^b^Case Nos. 7 and 8 are the present cases

The primary approach to treatment of Trousseau syndrome is to eliminate the causative tumor. This approach could be used for patients with cerebral infarction associated with adenomyosis, but the benign characteristics of the lesion and limited evidence for the cause make it hard to choose surgery as first-line treatment. A gonadotropin-releasing hormone (GnRH) agonist may be a treatment option, based on its effect of decreasing secretion of estrogen. However, side effects restrict the administration period of a GnRH agonist, and there is a report of a patient (Case No. 4 in Table 1) who had recurrent ischemic stroke after discontinuation of a GnRH agonist [8, 9]. Antithrombotic drugs are another treatment option. In patients with Trousseau syndrome, heparin, warfarin and other direct oral anticoagulants have been used to prevent thrombosis, although it is still unclear which drug is the most effective [10]. Anticoagulants and antiplatelet agents can also be used in patients with adenomyosis. In our patients, warfarin and rivaroxaban were administered and there have been no recurrent attacks. Long-term hormone replacement therapy may cause hypercoagulability in patients with adenomyosis, and discontinuation of this therapy in one reported case (Case No. 6, Table 1) did not lead to recurrence [11]. Overall, further studies are needed to clarify the mechanisms of development of cerebral infarction in patients with adenomyosis or other mucin-producing benign.

## Conclusions

In conclusion, we have reported two cases of cerebral infarction that seemed to be caused by adenomyosis. These cases suggest that cerebral infarction might develop in patients with a benign mucin-producing tumor, in addition to cases with a malignant tumor. Cerebral embolism in patients with adenomyosis is not common, but these patients may develop cerebral infarction due to hypercoagulability and elevated CA125. Therefore, we suggest inclusion of adenomyosis as a differential diagnosis in embolic stroke of an undetermined origin in middle-aged women.

## Abbreviations

CT: Computed tomography
DSA: Digital subtraction angiography
DWI: Diffusion-weighted imaging
ECG: Electrocardiogram
GnRH: Gonadotropin-releasing hormone
MCA: Middle cerebral artery
MRI: Magnetic resonance imaging
NIHSS: National Institutes of Health Score Scale
TEE: Transesophageal echocardiography
TTE: Transthoracic echocardiography
